# Perceived stigma and burden in natural caregivers of patients with schizophrenia

**DOI:** 10.1192/j.eurpsy.2021.1422

**Published:** 2021-08-13

**Authors:** F. Znaidi, R. Jenhani, A. Arousse, O. Moula, R. Ghachem

**Affiliations:** Manouba, Razi Hospital, Manouba, Tunisia

**Keywords:** schizophrénia, Natural caregiver, Stigma, Burden

## Abstract

**Introduction:**

Natural caregivers of patients with schizophrenia are often subjected to stigma by virtue of their association with patients. Affiliate stigma expose caregivers to community rejection, isolation and may have a negative impact on their psychological wellbeing.

**Objectives:**

This study aimed to assess perceived stigma and burden in a Tunisian population of natural caregivers of patients with schizophrenia and to identify risk factors for developing such disorders.

**Methods:**

We conducted a cross-sectional, descriptive and analytical study, including 80 natural caregivers of patients with schizophrenia. We used the Stigma Devaluation Scale (SDS) to assess stigma and the Zarit Burden Interview (ZBI) to evaluate burden.

**Results:**

The average age of natural caregivers was 55.7 years. The sex ratio (M/F) was 0.86. The mean score of perceived stigma in patients was 24.7. That of perceived stigma in caregivers was 15.34. Assessing the burden on caregivers estimated an average score of 58, corresponding to a severe burden. Medium to high burden was found in 78% of participants. Perceived stigma scores were significantly higher among illiterate caregivers, those linking schizophrenia to hereditary causes, among parents, and in case of daily contact with the patient. Scores of perceived stigma in caregivers were also significantly correlated with burden score.

**Conclusions:**

Natural caregivers of patients with schizophrenia are exposed to affiliate stigma and experience an important level of burden. Our findings emphasize the need to support natural caregivers of persons with schizophrenia and to develop strategies to combat stigmatization among patients as well as their natural caregivers.

